# Screening and Analysis of the Potential Bioactive Components in Rabbit Plasma after Oral Administration of Hot-Water Extracts from Leaves of *Bambusa textilis* McClure 

**DOI:** 10.3390/molecules17088872

**Published:** 2012-07-26

**Authors:** Jin Wang, Yong-De Yue, Feng Tang, Jia Sun

**Affiliations:** SFA Key Laboratory of Bamboo and Rattan Science and Technology, International Centre for Bamboo and Rattan, No. 8 Futong Dongdajie, Wangjing, Chaoyang District, Beijing 100102, China; Email: wangjin@icbr.ac.cn (J.W.); fengtang@icbr.ac.cn (F.T.); sunjia@icbr.ac.cn (J.S.)

**Keywords:** *Bambusa textilis* McClure, LC-Q-TOF-MS, chemical fingerprint, bioactive compounds in rabbit plasma, structure elucidation

## Abstract

*Bambusa textilis* McClure is a traditional Chinese medicinal plant belonging to the Bambusoideae subfamily and used to treat chronic fever and infectious diseases. To investigate the bioactive compounds absorbed in the rabbit blood after oral administration of hot-water extracts from the leaves of *B. textilis* McClure, a validated chromatographic fingerprint method was established using LC-Q-TOF-MS. Twenty compounds in bamboo leaves and three potential bioactive compounds in rabbit plasma were detected. Of the twenty detected compounds *in vitro*, fifteen of which were tentatively identified either by comparing the retention time and mass spectrometry data with that of reference compounds or by reviewing the literature. Three potential bioactive compounds, including (*E*)-*p*-coumaric acid, (*Z*)-*p*-coumaric acid, and apigenin-8-*C*-β-D-(2"-*O*-α-L-rhamnosyl)-gluco-pyranoside, were detected in both the leaves of *B. textilis* McClure and rabbit plasma. Of the three compounds, apigenin-8-*C*-β-D-(2"-*O*-α-L-rhamnosyl)glucopyranoside was identified based on its UV, MS, and NMR spectra. This study provides helpful chemical information for further pharmacology and active mechanism research on *B. textilis* McClure.

## 1. Introduction

Increasing research has focused on natural active compounds extracted from medicinal plants [1,2]. Bamboo leaves have been used in traditional Chinese medicine for treating fever and detoxification for over 1,000 years. It was found that extract of bamboo leaves has multiple biological activities, such as cancer preventive [3,4], anti-free radical and anti-oxidation [5,6], and can be used as a pharmaceutical intermediate and food additive. However, the bioactive compounds in bamboo leaves are not fully known. *B. textilis* McClure is one of the important medicinal bamboos types in China. Therefore, some strategies have to be designed for screening of bioactive compounds from bamboo leaves [7]. 

Only the compounds absorbed into the blood have the probability to become effective constituents. A plasma pharmacochemistry-based approach to screening potential bioactive components has been reported on some Chinese herbs and herbal preparations, such as DangGui [8], Huang Lian Jie Du Tang [9], ChanSu [10] and Yin Chen Hao Tang [11]. Plasma pharmacochemistry-based screening methods have provided significant advantages in quickly screening *in vivo* and giving a high probability of hitting active compounds [11]. Plasma pharmacochemistry techniques are proven to be effective tools for bioactive compound screening in medical plants [12]. The bioactive compounds can be ascertained by analyzing compounds absorbed in the blood after oral administration. 

In the present study, plasma pharmacochemistry techniques and a chromatographic fingerprint method using LC-Q-TOF-MS were performed to screen for bioactive compounds in *B. textilis* McClure. The potential bioactive compounds were ascertained by comparatively analyzing the chemical profiles of dosed rabbit plasma and extract of *B. textilis* McClure. The potential bioactive compounds were then identified based on their UV, NMR, and MS spectra.

## 2. Results and Discussion

### 2.1. HPLC Chromatograms of Plasma Samples

**Figure 1:**
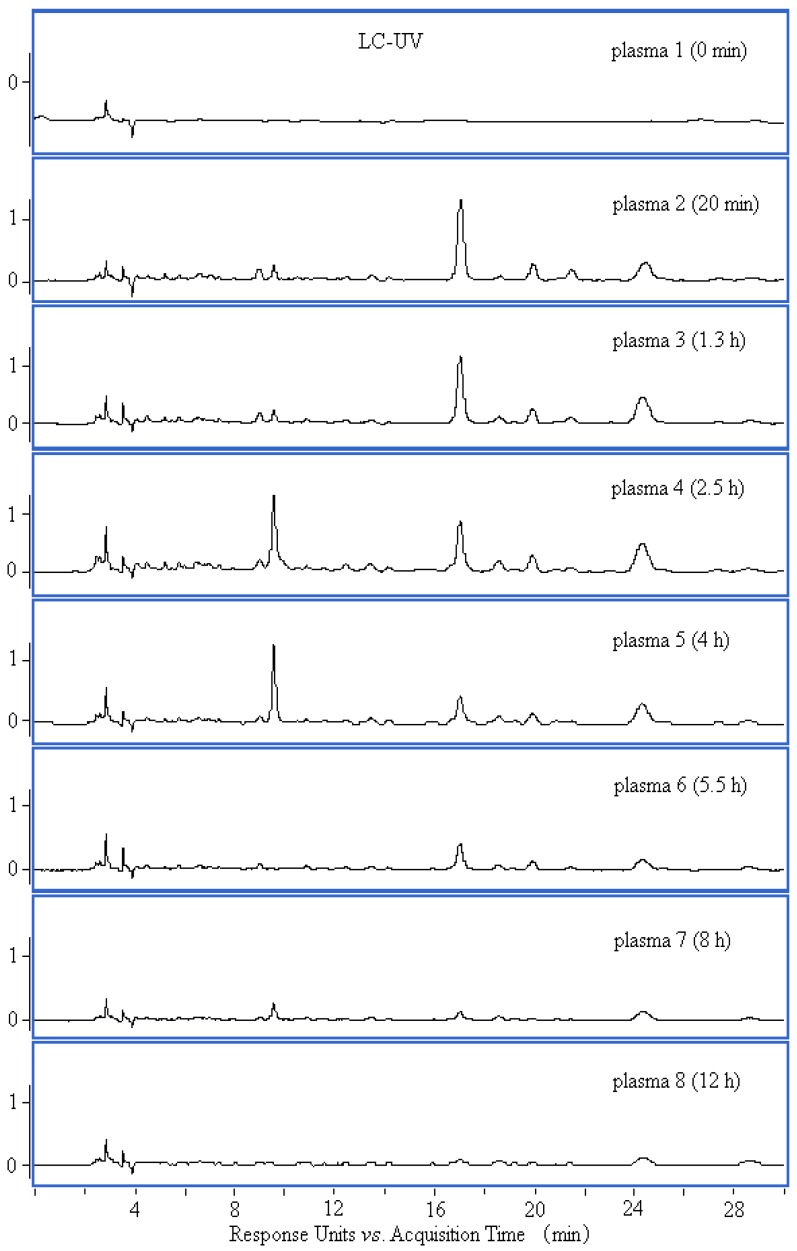
HPLC chromatograms (330 nm) of plasma samples taken at selected time points.

To achieve a good chromatographic resolution, chromatographic conditions were optimized. HPLC fingerprints of plasma samples collected at selected time points were obtained (Figure 1). As seen from Figure 1, a good separation of chemical constituents in plasma samples was achieved. It was found that more peaks with higher responses were detected in the chromatogram of plasma 4 at 2.5 h post-dose, whereas the chromatographic peaks of plasma 1 at 0 min pre-dose were not observed obviously at retention times from 5 min to 25 min. Thus, plasma 4 at 2.5 h post-dose was used to analyze the absorption compounds of hot-water extract from *B*. *textilis* McClure in rabbit.

### 2.2. Potential Bioactive Compounds Discovery

**Figure 2:**
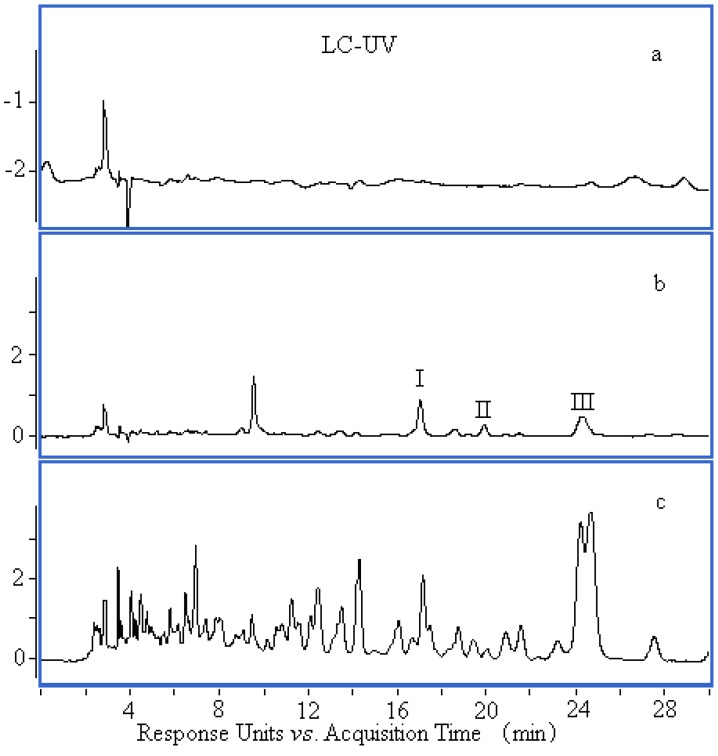
Chromatograms of control plasma (**a**), dosed plasma (**b**) and *B. textilis* extract (**c**).

Using LC-Q-TOF-MS, we acquired chromatographic profiles of control plasma, dosed plasma(2.5 h), and extract of *B*. *textilis* McClure (Figure 2). As shown in Figure 2, there were several peaks that appeared only in dosed plasma but do not appear in the chromatogram of control plasma. These compounds were absorbed into the blood and might be potential bioactive compounds from *B. textilis* extract. There are the absorbed components and metabolites in rabbit plasma. Except for the metabolites, the three selected peaks were clearly detected in both the leaves of *B. textilis* McClure and rabbit plasma. Thus, the peaks marked as I, II, and III in Figure 2(b) may be considered as the main bioactive compounds derived from *B*. *textilis* McClure.

### 2.3. LC-Q-TOF-MS Analysis of Hot-Water Extracts from *B. textilis* McClure and Plasma Sample

To validate the three proposed potential bioactive compounds derived from *B*. *textilis* McClure, both hot-water extract of *B*. *textilis* McClure and dosed plasma sample at 2.5 h were analyzed by LC-Q-TOF-MS. The MS data and the tentative identification results are shown in Table 1 and Table 2. 

LC-Q-TOF-MS technology can provide accurate mass, formula, UV spectra, retention time and main fragment information. As seen from Table 1, at least fifteen compounds were tentatively identified. Of the fifteen compounds, isoorientin, orientin, isovitexin, vitexin and *p*-coumaric acid were confirmed by comparing their accurate mass, UV spectra and retention times with those of standard compounds. Other compounds were identified by several means, utilizing their MS and MS/MS spectra, and comparing them with the literature data [13,14]. For example, a series of fragment ions of compound **5** are given in Table 1. In positive ion ESI mode, a [M+H]^+^ peak at *m/z* 611.1605 could confirm the molecular weight to be 610, so C_27_H_30_O_16_was possible molecular composition deduced by the MassHunter software. A fragment at *m*/*z* 449.1074 [(M+H)-162]^+^, corresponding to loss of one hexose unit, also appeared. The absence of the aglycone ion is consistent with a *C*-hexosyl unit. Compound **5** showed fragments ions at *m*/*z* 431.0957, *m*/*z* 413.0855 and *m*/*z* 329.0655 characteristic for *C*-hexosyl luteolin. Therefore, compound **5** was inferred to have the structure of *O*-hexosyl-*C*-hexosyl luteolin. 

**Table 1:** MS data and the identification results of the constituents from *B. textilis* by LC-Q-TOF-MS.

No.	RT(min)	[M+H]^+^	Main Fragments	Experimental *m*/*z*	Calculated *m*/*z*	Error		Formula	Tentative Identification
						mDa	ppm		
1.	3.65	151.0756	119.0496, 91.0547	150.0683	150.0681	−0.24	−1.59	C_9_H_10_O_2_	Phenolic acid
2.	7.84	611.1599	431.0969, 413.0870, 395.0752, 329.0654	610.1527	610.1534	0.71	1.17	C_27_H_30_O_16_	*O*-hexosyl-*C*-hexosyl luteolin
3.	8.80	209.1538	191.1425, 163.1483, 149.0958, 125.0953	208.1465	208.1463	−0.2	−0.96	C_13_H_20_O_2_	Phenolic acid
4.	9.80	535.2735	227.1631, 209.1527, 191.1416, 149.0947	534.2662	534.2676	1.44	2.69	C_25_H_42_O_12_	Unknown
5.	11.60	611.1605	449.1074, 431.0957, 413.0855, 329.0655	610.1532	610.1534	0.18	0.29	C_27_H_30_O_16_	*O*-hexosyl-*C*-hexosyl luteolin
6.	12.31	595.1661	415.1013, 397.0907, 379.0807, 337.0697	594.1588	594.1585	−0.32	−0.54	C_27_H_30_O_15_	Di-O, *C*-hexosyl-apigenin
7.	12.56	581.1497	449.1070, 431.0964, 413.0858, 329.0650	580.1424	580.1428	0.4	0.69	C_26_H_28_O_15_	*O*-pentosyl-6-*C*-hexosyl luteolin
8.	13.38	565.1547	547.1427, 511.1217, 379.0807, 325.0701	564.1474	564.1479	0.46	0.82	C_26_H_28_O_14_	*C*-hexosyl-*C*-pentosyl-apigenin
9.	14.50	595.1653	449.1077, 431.0965, 413.0861, 329.0656	594.1580	594.1585	0.47	0.8	C_27_H_30_O_15_	O,*C*-dideoxyhexosyl-luteolin
10.	14.61	449.1074	431.0959, 413.0860, 395.0757, 329.0651	448.1001	448.1006	0.45	1.01	C_21_H_20_O_11_	Isoorientin
11.	15.06	385.1633	325.1366, 217.0861, 181.0847, 167.0697	384.1560	384.1573	1.27	3.32	C_22_H_24_O_6_	Flavonoid
12.	16.31	449.1078	431.0969, 413.0874, 383.0746, 329.0652	448.1005	448.1006	0.05	0.11	C_21_H_20_O_11_	Orientin
13.	17.61	165.0548	147.0448, 119.0494	164.0475	164.0473	−0.18	−1.08	C_9_H_8_O_3_	(*E*)*-p*-coumaric acid
14.	20.40	165.0546	147.0440, 119.0490	164.0469	164.0473	−0.4	2.46	C_9_H_8_O_3_	(*Z*)-*p*-coumaric acid
15.	21.53	147.044	119.0490, 91.0545	146.0367	146.0368	0.08	0.54	C_9_H_6_O_2_	Coumarin
16.	21.88	565.1552	433.1116, 415.1013, 337.0704, 313.0707	564.1479	564.1479	−0.03	−0.05	C_26_H_28_O_14_	*O*-pentosyl-*C*-hexosyl-apigenin
17.	22.81	433.1118	415.1008, 397.0900, 367.0819, 313.0696	432.1046	432.1056	1.09	2.51	C_21_H_20_O_10_	Vitexin
18.	24.44	197.1171	179.1058, 161.0958, 135.1165, 107.0852	196.1098	196.1099	0.11	0.57	C_11_H_16_O_3_	Phenolic acid
19.	24.80	579.1711	433.1130, 415.1024, 397.0919, 313.0710	578.1639	578.1636	−0.31	−0.54	C_27_H_30_O_14_	*O*-hexosyl-*C*-hexosyl-apigenin
20.	25.09	433.1125	415.1020, 379.0810, 337.0704, 313.0701	432.1053	432.1056	0.38	0.89	C_21_H_20_O_10_	Isovitexin

The three potentially bioactive compounds in Table 2 were found to have the same MS data as No. **13**, **14** and **19** in Table 1. The results further confirmed that the three compounds were derived from *B*. *textilis* McClure, so we next need to further identify the chemical structure of the potential bioactive compounds.

**Table 2:** MS data and the identification results of three compounds from dosed plasma (2.5 h) by LC-Q-TOF-MS.

Compound	RT (min)	[M+H]^+^	Main fragments	Experimental *m*/*z*	Calculated *m*/*z*	Error		Formula	Tentative Identification
						mDa	ppm		
Ⅰ	17.68	165.0543	147.0430, 119.0490, 91.0546	164.0470	164.0473	0.34	2.06	C_9_H_8_O_3_	( *E*)-*p*-coumaric acid
Ⅱ	20.35	165.0545	147.0445, 119.0491, 91.0548	164.0473	164.0473	0.09	0.52	C_9_H_8_O_3_	( *Z*)-*p*-coumaric acid
Ⅲ	24.81	579.1705	433.1129, 415.1021, 313.0703	578.1632	578.1636	0.33	0.57	C_27_H_30_O_14_	*O*-hexosyl-*C*-hexosyl-apigenin

### 2.4. Structure Identification of Three Potential Bioactive Compounds

**Figure 3:**
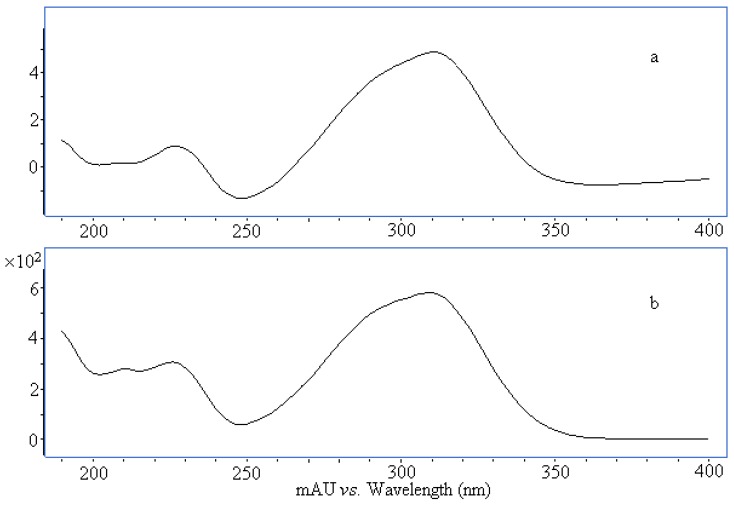
Comparison UV spectra of compound Ι (**a**) and *p*-coumaric acid (**b**).

**Figure 4:**
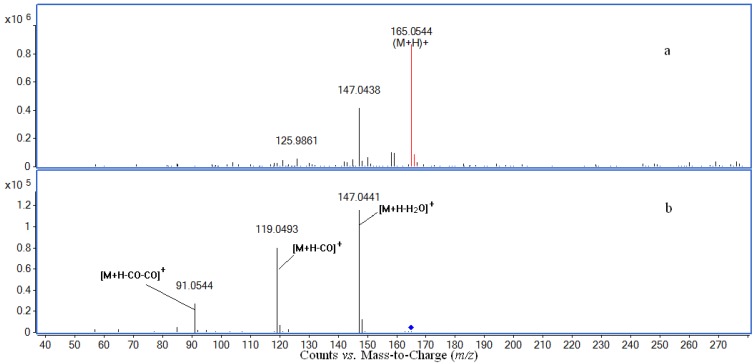
ESI-MS (**a**) and MS/MS (**b**) spectra of *p*-coumaric acid.

**Figure 5:**
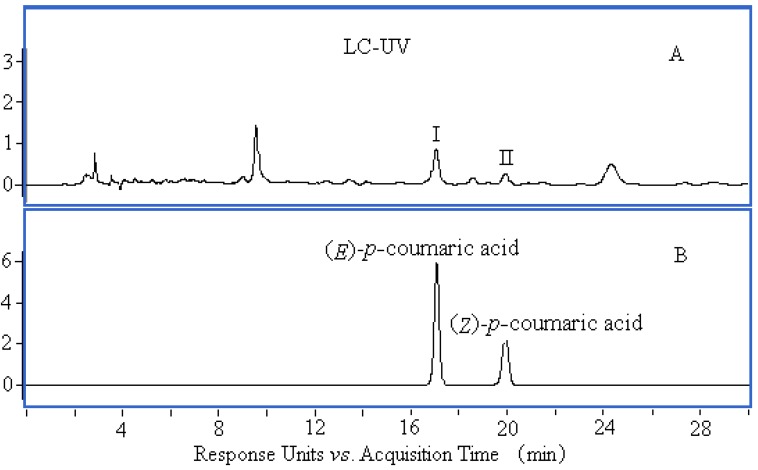
HPLC chromatograms of plasma sample (**A**) and *p*-coumaric acid standards (**B**).

**Figure 6:**
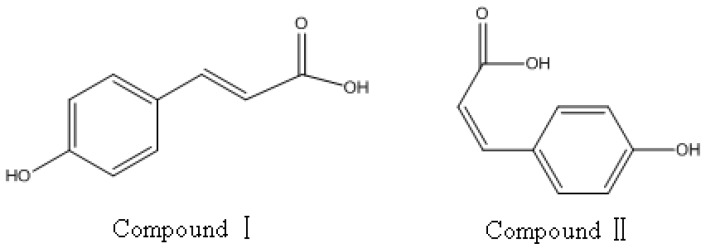
Structures of compound I and compound II.

Compounds I and II in Table 2 were identified by comparing their UV spectrum, accurate mass, main fragment ions and retention time with those of (*E*)-*p*-coumaric acid and (*Z*)-*p*-coumaric acid standards (Figure 3, Figure 4 and Figure 5). The chemical structures of compounds Ι and ΙΙ are shown in Figure 6.

### 2.5. UV Spectrum of Compound III

**Figure 7:**
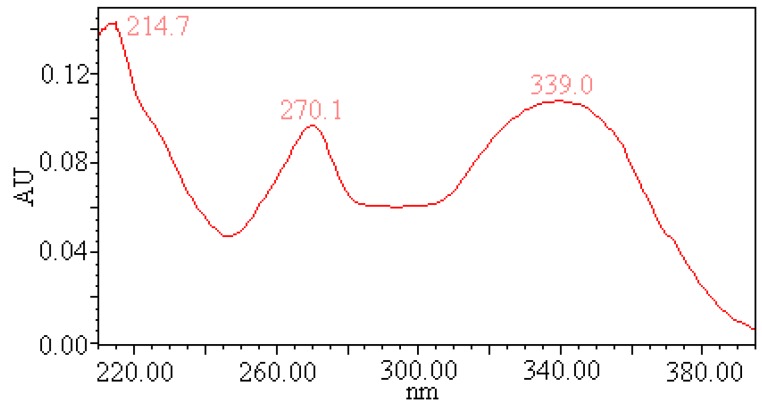
UV spectrum of compound III.

The UV spectrum of Compound III was a typical flavonoid UV spectrum according to its two major absorption bands in the UV region, band I (300–400 nm) and band II (220–280 nm) (Figure 7). Flavonoids are a large group of polyphenolic compounds possessing a basic flavan nucleus with two aromatic rings (the A and the B rings) interconnected by a three-carbon-atom heterocyclic ring (the C ring). The band I of compound III appear at 339.0 nm indicating that the compound III belongs to the flavone family unsubstituted at 3-position. The UV spectrum of the compound III showed a single peak at 270.1 nm, indicating that B ring contains only a 4′-OH group.

### 2.6. NMR Analysis of Compound III

The ^1^H-NMR spectrum of Compound III established the presence of five aromatic protons at δ 6.47 (1H, s), δ 7.81 (2H, d, *J =* 7.5, H-2′, 6′) and δ 6.81 (2H, d, *J =* 7.2, H-3′, 5′). Of these protons, the appearance of two doublets and their coupling constants values were δ 7.81 (*J =* 7.5) and δ 6.81 (*J =* 7.2), which were clearly assignable to ring B protons at H-2′, H-6′ and H-3′, H-5′, respectively. A single sharp peak at δ 6.72 ppm was assigned to the H-3 proton of the C ring. The ^1^H-NMR spectrum of the compound exhibited signals at δ 4.52 (*J =* 9.9) applicable for sugar anomeric protons suggesting the presence of a β-glucopyranoside [15]. According to its ^13^C-NMR spectrum, a high-field signal at δ 17.9 (C-6′′′) was indicative for the presence of α-rhamnosyl. A downfield signal at δ 182.6 was clearly assigned to the carbonyl carbon C-4 of the pyrone ring. The three downfield signals appearing at δ 161.6, δ 163.1 and δ 156.7 were assigned to the C-5, C-7 and C-4′ carbon atoms bearing hydroxyl groups. Furthermore, a signal at δ 80.5 suggested that a rhamnosyl unit was attached to C-2′′.

### 2.7. LC-Q-TOF-MS Analysis of Compound III

**Figure 8:**
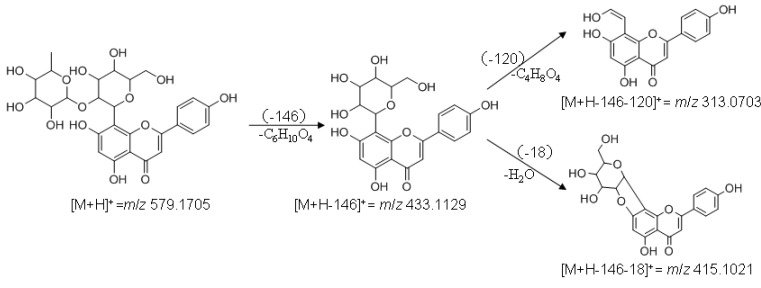
Main fragmentation pathways of compound III.

Compound III had a pseudomolecular ion at *m*/*z* 579.1705 [M+H]^+^. A molecular formula of C_27_H_30_O_14_ (calc. 578.1636) was generated using the MassHunter quantitative analysis software. A fragment at *m*/*z* 433.1129 [M+H-146]^+^, corresponded to loss of one deoxyhexose. Thus, compound III could have a deoxyhexosyl unit in the terminal position of a disaccharide unit. The presence of the ion at *m*/*z* 313.0703 [M+H-146-120]^+^ and the absence of the fragment [M+H-146-60]^+^ indicated a hexose as the *C*-glycosylation sugar. As analyzed above, the structure of compound III was determined as apigenin-8-*C*-β-D-(2"-*O*-α-L-rhamnosyl)-glucopyranoside, which was also confirmed by its MS fragmentation pathways (Figure 8).

Bamboo leaves are rich in flavonoids and the main functional components of extract from bamboo leaf are flavone *C*-glycosides [16,17]. According to the literature flavone *C*-glycosides, including isoorientin, orientin, vitexin and isovitexin, were poorly absorbed in the gastrointestinal tract of rat. Flavone *C*-glycosides largely reach the colon where they can be degraded into various aromatic acid metabolites by the microflora. The flavone *C*-glycosides cannot be absorbed in the rats’ blood after oral administration, whereas *p*-coumaric acid can be absorbed in blood [18]. The ability of *p*-coumaric acid to protect rat’s heart against doxorubicin-induced oxidative stress has been proved [19]. In this study, apigenin-8-*C*-β-D-(2"-*O*-α-L-rhamnosyl)-glucopyranoside belonging to the *C*-glycosides class was absorbed in rabbit blood. The absorption mechanism *in vivo* should be investigated in further research. This study provides helpful chemical information for further our understanding of the pharmacology and active mechanism research of *B. textilis* McClure.

## 3. Experimental

### 3.1. General

Liquid chromatography/quadrupole time-of-flight mass spectrometry (LC-Q-TOF-MS) analysis was performed on an Agilent 1290 series LC system coupled with a Q-TOF (model 6540, Agilent Technologies, Santa Clara, CA, USA) and equipped with a diode array detector (DAD). The system was controlled under MassHunter B.04 software. Purification of bioactive compounds was performed with a Gilson preparative HPLC GX-281/322/156 system (Gilson, Middleton, WI, USA), using a C18 column (250 × 20 mm I.D., 5 µm, Hydrosphere, YMC Japan, Kyoto, Japan). The obtained compounds were dissolved in 0.5 mL of DMSO-*d*_6_ and the solution was filled into a NMR tube. NMR analysis was carried out using a Bruker 300 MHz spectrometer operating at 300 (^1^H) or 75 MHz (^13^C), respectively. Chemical shifts were reported in ppm on δ scale, and the coupling constants (*J*) were measured in Hertz (Hz). The compounds were identified by a combination of spectroscopic methods (^1^H-, ^13^C-NMR), ESI-MS and comparison with the literature data.

### 3.2. Materials

The bamboo leaves of *B. textilis* McClure were collected from the bamboo garden of the Jiangxi Academy of Forestry, Nanchang, China. The species were authenticated by Professor Jiu-Sheng Peng from the Jiangxi Academy of Forestry. Bamboo leaves were dried in the shade, ground to powder, and stored at –20 °C. 

### 3.3. Chemicals

The HPLC-grade acetonitrile and methanol were purchased from Fisher Scientific (Fair Lawn, NJ, USA). Acetic acid and (*E*)-*p*-coumaric acid were purchased from Sigma-Aldrich (St. Louis, MO, USA). (*Z*)-*p*-coumaric acid was purchased from J&K Scientific Ltd. (Beijing, China). Water was purified with an ultrapure water system (Purelab Plus, Pall, Port Washington, NY, USA). Analytical-grade chemical was obtained from Beijing Chemical Works (Beijing, China). The standards of isoorientin, orientin, isovitexin and vitexin were purchased from Shanghai Winherb Medical S & T Development Co., Ltd. (Shanghai, China).

### 3.4. Preparation of Hot-Water Extracts of *B. textilis* McClure

The dried bamboo leaf powder (100 g) was extracted by percolation with water (600 mL) and the solution was heated to reflux and boiled gently for 20 min. The cooled solution was filtered and the residue of the bamboo leaves was carefully washed twice with water (600 mL). The combined extracts were concentrated under reduced pressure at 40 °C. The extracts were suspended in water (100 mL) and stored at 4 °C before use.

### 3.5. Preparation of Plasma Samples

A rabbit (3 kg) was obtained from the SFA Key Laboratory of Bamboo and Rattan Science and Technology (Beijing, China). The rabbit was fasted 16 h before the experiment and water was taken freely. Hot-water extracts from *B. textilis* McClure (100 g of bamboo leaves/100 mL of water) was administered orally to the rabbit at a single dose of 33.3 g/kg. Blood samples (2.5–3 mL) were collected in heparinized tubes pre-dose (0 min) and at selected time points (20 min, 1.3 h, 2.5 h, 4 h, 5.5 h, 8 h and 12 h) post-dose. The heparinized tubes were shaken gently as soon after collection as possible to prevent clotting. Then the blood samples were centrifuged at 3,000 rpm for 10 min. The supernatant layer of plasma (0.5 mL) was placed into a 5 mL polypropylene tube. Methanol (2 mL) was added to the tube to deposit proteins. Each tube was vortexed for 30 s and left for 12 h at –18 °C. Then plasma samples were centrifuged at 13,000 rpm for 10 min. The supernatant was transferred to a round-bottom flask, and evaporated to dryness at 40 °C in a rotary vacuum evaporator (Rotary Evaporator, EYELAN-1001D-W, Tokyo, Japan). The residue was reconstituted in 0.5 mL of methanol and then filtered through a 0.22 μm membrane. Experiments were carried out in accordance with the Guide for the Care and Use of Laboratory Animals [8,20].

### 3.6. LC-Q-TOF-MS Analysis

LC separation was performed using a C18 column (4.6 × 250 mm, 5 µm, YMC pack R&D ODS-A, YMC Japan) at 30 °C. The solvent system consisted of a mixture of water with 0.5% (v/v) acetic acid and acetonitrile (85/15, v/v) at a flow rate of 1.0 mL/min. The LC effluent was split using a T-splitter to produce a flow of 0.25 mL/min [21]. 

The TOF/MS analysis worked using positive mode. Mass spectra were acquired in the range of 100 to 1,000 *m*/*z* for MS^1^ and 20 to 1,000 *m*/*z* for MS^2^. The MS^n^ data were collected in an auto MS/MS mode. The collision-induced dissociation (CID) voltage was set at 20 V. The conditions of ESI source were as follows: drying gas (N_2_) flow rate, 8 L/min; drying gas temperature, 350 °C; nebulizer, 45 psig; capillary voltage, 4,000 V; skimmer voltage, 65 V; fragmentor voltage, 175 V; sheath gas temperature, 350 °C; nozzle voltage, 1,000 V. During the analysis, two reference masses were used: 121.0509 (C_5_H_4_N_4_) and 922.0098 (C_18_H_18_O_6_N_3_P_3_F_24_). These masses are continuously infused to the system to allow constant mass correction. The chemical formula of the selected peaks were calculated by the accurate mass of the precursor and product ions. The formula predictor was set as follows: C (0-60), H (0-120), O (0-30). Other elements such as P, S, F and Cl were not considered, since they are rarely present in the components of bamboo leaves.

### 3.7. Isolation of Active Compound by Preparative HPLC

Powdered leaves (100 g) of *B. textilis* McClure were extracted again as above. After removing the solvent under reduced pressure and freeze-drying, the combined extracts were concentrated and yielded 18 g of a crude extract. The extract was suspended in water/acetonitrile (100 mL, 85/15, v/v) and filtered through a 0.45 μm membrane to remove the insoluble material. This crude solution was purified on a preparative HPLC system. The mobile phase consisted of H_2_O (solvent A) and ACN (solvent B). The gradient elution program was as follows: 0–3 min, 0% B; 3–7 min, 0–15% B; 7–45 min, 15% B; 45–50 min, 15–50% B; 50–52 min, 50–15% B; 52–60 min, 15% B. The flow rate was set at 10 mL/min with UV detection at 342 nm to yield compound III (6.8 mg).

### 3.8. Analytical Data

*Apigenin-8-C-β-D-(2"-O-α-L-rhamnosyl)-glucopyranoside* (**III**). Yellowish solid. UV λ_max_(ACN-H_2_O) 270, 339 nm; HRESIMS *m*/*z* 579.1705 [M+H]^+^ (calc. for C_27_H_30_O_14,_ 579.1708); ^1^H-NMR (DMSO-*d*_6_) δ: 6.72 (1H, s, H-3), 6.47 (1H, s, H-6), 7.81 (2H, d, *J=* 7.5, H-2′, 6′), 6.81 (2H, d, *J=* 7.2, H-3′, 5′), 4.52 (1H, d, *J=* 9.9, H-1′′), 4.97 (1H, s, H-1′′′), 3.40–4.50 (13H, m, Sugar); ^13^C-NMR (DMSO-*d*_6_) δ: 163.8 (C-2), 103.1 (C-3), 182.6 (C-4), 161.6 (C-5), 94.7 (C-6), 163.1 (C-7), 109.5 (C-8), 156.7 (C-9), 109.0 (C-10), 121.5 (C-1′), 128.8 (C-2′), 116.2 (C-3′), 156.7 (C-4′), 116.2 (C-5′), 128.8 (C-6′), 75.0 (C-1′′), 80.5 (C-2′′), 76.2 (C-3′′), 71.7 (C-4′′), 81.9 (C-5′′), 62.2 (C-6′′), 103.1 (C-1′′′), 71.7 (C-2′′′), 71.1 (C-3′′′), 71.4 (C-4′′′), 72.0 (C-5′′′), 17.9 (C-6′′′).

## 4. Conclusions

LC-Q-TOF-MS analysis coupled with a plasma pharmacochemistry method was applied to analyze the bioactive compounds in the leaves of *B. textilis* McClure. A total of 20 compounds were detected in bamboo leaves and three potential bioactive compounds detected in rabbit plasma were the original form of the compounds detected *in vitro*. Based on their UV, MS, and NMR spectra, the potential bioactive compounds were identified as follows: (*E*)-*p*-coumaric acid, (*Z*)-*p*-coumaric acid, and apigenin-8-*C*-β-D-(2"-*O*-α-L-rhamnosyl)-glucopyranoside. This approach provides a strategy for screening and characterizing bioactive compounds in medicinal bamboo. 

## Supplementary Materials

Supplementary materials can be accessed at: http://www.mdpi.com/1420-3049/17/8/8872/s1.
