# Supplementary material for: Screening and Analysis of the Potential Bioactive Components in Rabbit Plasma after Oral Administration of Hot-Water Extracts from Leaves of Bambusa textilis McClure
Source: Molecules. 2012 Jul 26;17(8):8872–85. doi: 10.3390/molecules17088872 (PMC6268862; doi:10.3390/molecules17088872)

Molecules **2012**, *17*, 8872-8885; doi:10.3390/molecules17088872

OPEN ACCESS

molecules

ISSN 1420-3049

www.mdpi.com/journal/molecules

Article

Screening and Analysis of the Potential Bioactive Components in Rabbit Plasma after Oral Administration of Hot-Water Extracts from Leaves of *Bambusa textilis* McClure

Jin Wang, Yong-De Yue *, Feng Tang and Jia Sun

SFA Key Laboratory of Bamboo and Rattan Science and Technology, International Centre for Bamboo and Rattan, No. 8 Futong Dongdajie, Wangjing, Chaoyang District, Beijing 100102, China; E-Mails: wangjin@icbr.ac.cn (J.W.); fengtang@icbr.ac.cn (F.T.); sunjia@icbr.ac.cn (J.S.)

* Author to whom correspondence should be addressed; E-Mail: yueyd@icbr.ac.cn; Tel./Fax: +86-10-8471-3741.

Received: 8 June 2012; in revised form: 17 July 2012 / Accepted: 18 July 2012 / Published: 26 July 2012

Supplementary Materials

Figure 1. ^{1}H-NMR of compound III.

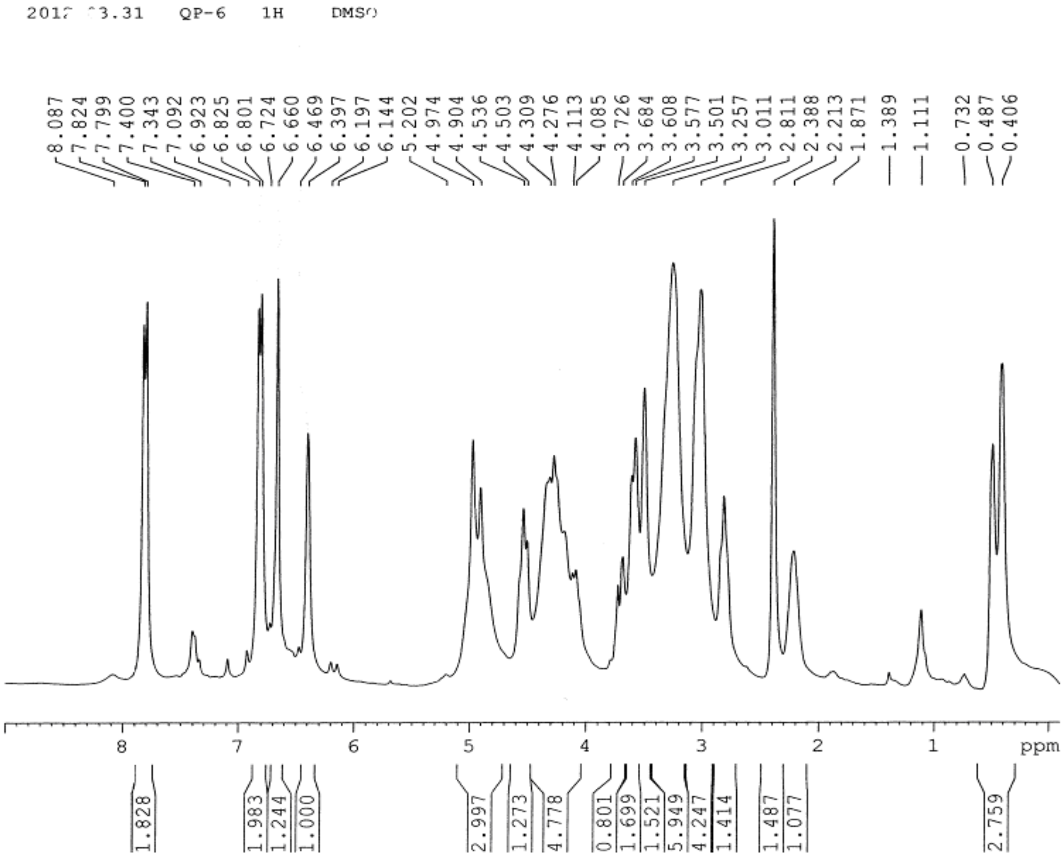

Figure 2. ^{13}C-NMR of compound III.

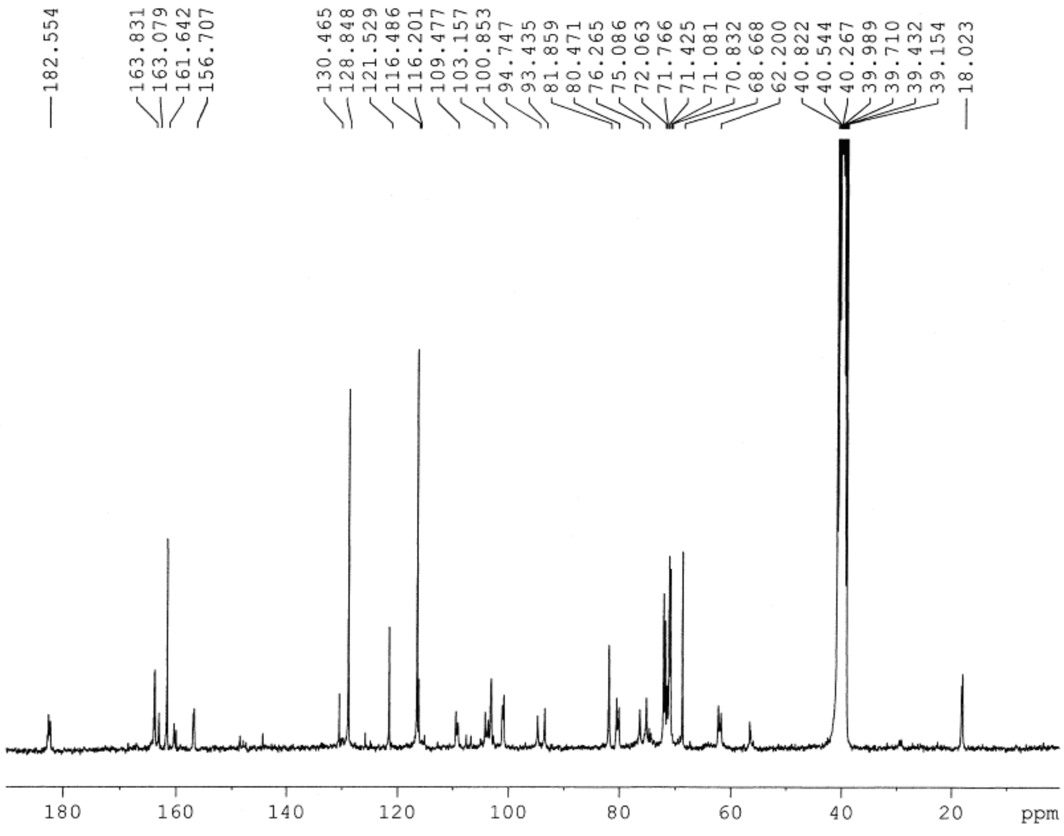

Figure 3. DEPT 135 NMR spectrum for compound III.

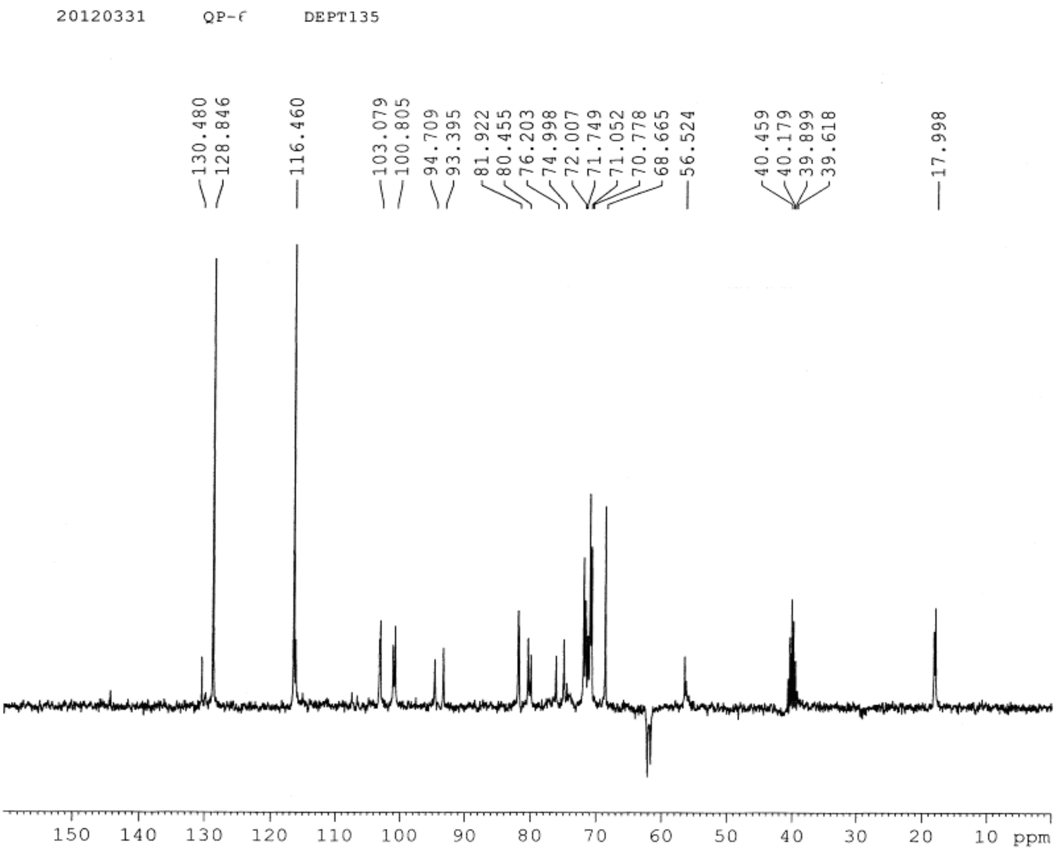

Figure 4. DEPT 90 NMR spectrum for compound III.

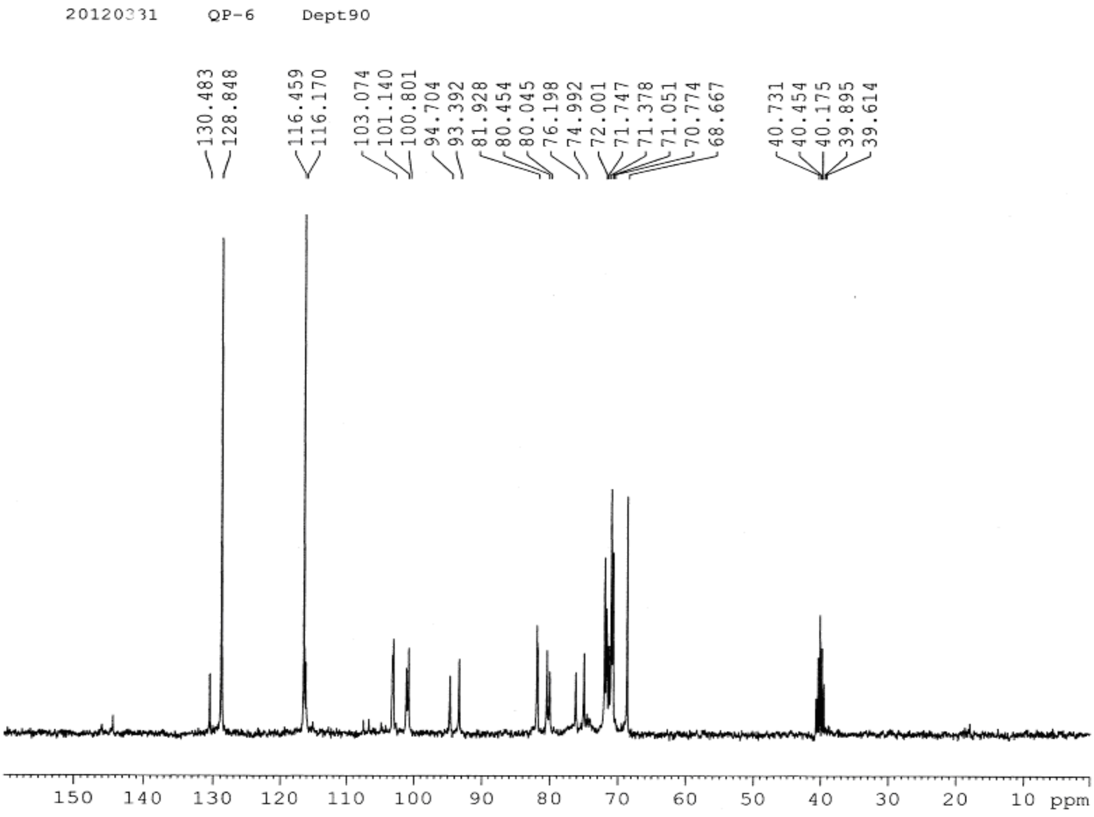

Supplement: Supplementary file 1 [file molecules-17-08872-s001.pdf]
